# Influence of age on molecular characteristics and rheological behavior of nopal mucilage

**DOI:** 10.1002/fsn3.2629

**Published:** 2021-10-13

**Authors:** Francisco Rodríguez‐González, José Pérez‐González, Cesar Nadem Muñoz‐López, Silvia Viridiana Vargas‐Solano, Benjamín M. Marín‐Santibáñez

**Affiliations:** ^1^ Departamento de Biotecnología Centro de Desarrollo de Productos Bióticos Instituto Politécnico Nacional Yautepec Morelos México; ^2^ Laboratorio de Reología y Física de la Materia Blanda Escuela Superior de Física y Matemáticas Instituto Politécnico Nacional Ciudad de México México; ^3^ Escuela Superior de Ingeniería Química e Industrias Extractivas Instituto Politécnico Nacional Ciudad de México México

**Keywords:** age, molecular weight, mucilages, *Opuntia ficus‐indica* (nopal), rheological behavior

## Abstract

Nopal mucilages are of interest due to a variety of potential applications, which include their use as thickeners and rheological modifiers. In this work, changes in molecular characteristics of nopal mucilages with their age and its influence on the rheological behavior of their solutions were analyzed by light scattering, high performance liquid chromatography, a colorimetric method, and linear viscoelastic rheometrical measurements. For this, mucilages obtained from cladodes from *Opuntia ficus‐indica* with different ages, namely 20, 80, and 600 days, respectively, were extracted using water as solvent and then subjected to freeze‐drying. The weight‐average molecular weight (*M*_w_) of the mucilages was found to increase along with the age; meanwhile, the concentration of uronic acids increased and the galactose, rhamnose, and xylose contents decreased. Increasing *M*_w_ with age resulted in enhanced viscoelastic behavior of solutions, namely, higher viscosity and elasticity at lower mucilage concentrations. Also, along with increasing *M*_w_, decrease in neutral sugar contents and increase of pectic compounds (uronic acids) and Ca^+^ cations in mucilages with age promoted formation of weak gels. Overall, the molecular weights and rheological behaviors reported in this work for nopal mucilages are comparable to those of widely used thickeners such as xanthan and guar gums, which suggest their use in similar applications regarding its age.

## Graphical abstract

The manuscript presents an assessment of the effects of age of nopal on some of the molecular characteristics of its mucilages, such as molecular weight and sugar contents, as well as their influence on the rheological behavior of their solutions.

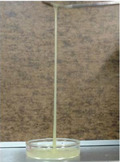

## 1 INTRODUCTION

The *Opuntia* genus of the cactus family is cultivated in different countries for alimentary and industrial purposes. This type of cactus is composed of flat joints with paddle‐like cladodes or nopals, which are edible and contain multiple channels or conduits in its structure, these are filled with a branched high molecular weight polysaccharide known as mucilage (Trachtenberg & Mayer, 1981). The mucilage from nopal is constituted by a large variety of neutral sugars including fructose, glucose, d‐xylose, l‐rhamnose, l‐arabinose, and d‐galactose, among others, as well as by some acid sugars such as galacturonic, glucuronic, and uronic acids (Forni et al., 1994; McGarvie & Parolis, 1979, 1981a,b; Trachtenberg & Mayer, 1981). The composition of sugars in the mucilage depends on certain factors such as the variety of the cladodes and their age (Ribeiro et al., 2010), as well as on the type and pH of the soil (McGarvie & Parolis, 1979, 1981a,b; Trachtenberg & Mayer, 1981). Also, the amounts of neutral and/or acid sugars affect the molecular weight of this biopolymer (Cárdenas et al., 1997; Majdoud et al., 2001; Médina‐Torres et al., 2000; Trachtenberg & Mayer, 1981).

The mucilage from nopal is water soluble and has the capacity to form viscoelastic solutions and gels, depending on its molecular weight and concentration. Therefore, this polysaccharide is often considered as a hydrocolloid with potential applications as thickener (Bernardino‐Nicanor et al., 2015), as a functional ingredient in foods (Dick et al., 2020; Du Toit et al., 2019), as well as an emulsion stabilizer in the cosmetic and pharmaceutical industries (Du Toit et al., 2020; Quinzio et al., 2018). In addition, nopal mucilages have been used in the preparation of biodegradable packings and coatings to increase the shelf life of vegetables and fruits (Allegra et al., 2017; Gheribi et al., 2018), meanwhile as the elastic properties of their solutions (film forming capabilities) are well known, nopal mucilages are often used as binders in rustic paints.

Despite the large amount of investigations about the physical–chemical properties of mucilage from *O. ficus‐indica*, most of them have focused on the physiological properties of the plant, the variety and age of the cladodes, as well as the neutral and acid sugars’ effects (Contreras‐Padilla et al., 2016; Ribeiro et al., 2010). So far, there is a dearth of studies on the influence of these characteristics of the plant on the rheological behavior of its mucilages in solutions. Trachtenberg and Mayer (1982) reported one of the earliest analyses of the rheological behavior of nopal mucilage solutions. These authors studied solutions of mucilage (weight‐average molecular weight, *M*_w_ = 4.3 × 10^6^ g/mol, for simplicity, hereafter we will refer only to molecular weight) with different concentrations of calcium chloride (CaCl_2_) and found that their viscosities decreased with increasing the concentration of the salt or ionic strength. Later on, Cárdenas et al. (1997) carried out a rheological characterization of nopal mucilage solutions (*M*_w_ = 3.4 × 10^6^ g/mol) at different concentrations under oscillatory and steady shear flow. The authors reported viscoelastic behavior and a pronounced shear‐thinning with increasing mucilage concentration. Médina‐Torres et al. (2000) analyzed the effect of concentration, temperature, and ionic strength on the viscoelastic properties of solutions of nopal mucilage. The solutions exhibited shear‐thinning behavior and the viscosity decreased with increasing the ionic strength. Also, the elasticity of solutions, assessed via the first normal stress difference, decreased with increasing the mucilage concentration at a given shear stress.

Separately, Majdoud et al. (2001) investigated the influence of the degree of purification of nopal mucilage, with and without proteins, respectively, as well as the addition of mono (LiNO_3_, lithium nitrate) and divalent (CaCl_2_) cations on the rheological properties of mucilage solutions. These authors reported shear‐thinning behavior of the solutions and higher viscosity for the protein‐free solution mucilage at a given concentration and shear rate. Majdoud et al. also suggested the validity of the Cox–Merz rule and their viscosity data were well fitted by the Carreau model. Finally, these authors reported a decrease in viscosity of the solutions with cations as compared to a solution without cations, which was attributed to the screening of electric charges in the macromolecules. Cárdenas et al. (2008) studied mucilage solutions with different concentrations of CaCl_2_ by dynamic oscillatory shear flow in a temperature range from 5 to 85°C and observed the formation of gel‐like structures when decreasing the temperature, as well as an increase in the gelation temperature with increasing the concentration of CaCl_2_.

León‐Martínez et al. (2011) prepared solutions with mucilages obtained from 13‐month‐old cladodes by two different processes, namely, spray‐ and freeze‐drying. The authors noted that all the solutions exhibited shear‐thinning behavior, but the solutions with the mucilage obtained by freeze‐drying showed higher viscosities than those with the mucilage obtained by spray drying for a given shear rate and concentration. Also, by using dynamic oscillatory measurements, these authors showed that solutions became gel like at lower concentrations when using the freeze‐dried mucilage. This behavior was attributed to damage on the macromolecules (likely, a decrease in molecular weight) due to the spray drying process. Finally, Contreras‐Padilla et al. (2016) analyzed the age effect (50, 100, and 150 days) of cladodes of *O. ficus‐indica* on the rheological behavior of their solutions and reported a shear‐thinning behavior as well as an increase in viscosity with increasing the mucilage concentration and age of the cladodes. In addition, these authors reported gel‐like behavior for all the solutions with concentrations between 3% and 5% w/v; the older the mucilage, the more elastic the solutions. These results were suggested to be due to changes in the chemical composition and structure of the mucilages along with the age of the cladodes.

From the aforementioned references, it seems that apart from the influence of salts, which alter the ionic strength of solutions, the rheological behavior of nopal mucilage solutions is mainly dependent on the molecular weight of the mucilage and this, in turn, depends on the age of the plant. However, to our knowledge, there is no systematic study of the change in molecular characteristics of mucilage from cladodes of *O. ficus*‐*indica* with their age and its effect on the rheological behavior of their solutions. Therefore, the aim of this work was to evaluate the changes in molecular characteristics of mucilages from cladodes of *O. ficus*‐*indica* with their age and its influence on the rheological behavior of their solutions. With this purpose, we have determined the molecular weight (*M*_w_) of mucilages from cladodes with different ages by static light scattering (SLS), their sugar contents using high performance liquid chromatography (HPLC), and the colorimetric method using carbazole, to finally determine their influence on the rheological behavior of their solutions via dynamic oscillatory shear flow rheometry. Here, we show for the first time that the *M*_w_ of the mucilages increases along with their age, meanwhile the concentration of uronic acids increases and the galactose, rhamnose, and xylose contents decrease. Increasing *M*_w_ with age results in enhanced viscoelastic behavior of solutions, that is, higher viscosity and elasticity at lower mucilage concentrations. Also, along with increasing *M*_w_, decrease in neutral sugar contents and increase of pectic compounds (uronic acids) and Ca^2+^ cations in mucilages with age promote the formation of weak gels.

## 2 MATERIALS AND METHODS

### 2.1 Collection of cladodes and extraction of mucilages

Cladodes from *O. ficus‐indica* with 20, 80, and 600 days of age identified as tender (*T*), young (*Y*), and mature (*M*), respectively, were harvested in the same farming area, that is, the region of Santa Catarina, Tepoztlán, Morelos, México. The selection and collection of the cladodes was random from different plants. Since, the climate, temperature, and harvesting time of the cladodes may affect the physical–chemical properties of the mucilages and their rheological behavior in solution (Du Toit et al., 2020), the cladodes were harvested in just one season, that is, during the spring in Morelos, in the months of March and April, systematically early in the morning (at 6:00 a.m.) when the average temperature is around 12°C. The cladodes were harvested by weight, namely, 4 kg of each type, which resulted in 58 ± 1, 28 ± 1, and 16 ± 2 pieces of *T*, *Y*, and *M* cladodes, respectively. Afterward, the cladodes were manually cleaned from spines, washed with fresh‐water and liquid soap, and finely sliced for mucilage extraction.

Mucilage extraction followed the procedure proposed by Sepúlveda et al. (2007) using distilled water as solvent. For this, the sliced cladodes were introduced in beakers with water in a 1:1 w/v ratio and heated at 40 ± 2°C while stirring at 300 rpm on a magnetic stirrer for 4 hr. The solutions with the extracted mucilages were filtered using a #40 sieve (~420 μm) and refrigerated at 4°C for 18 hr. Finally, these solutions were freeze‐dried (Genesis 12 SE, VirTis, SP Industries) under high vacuum (0.04 mbar) for 6 days to obtain the dried mucilage powders.

The reason for using water in mucilage extraction was to obtain an economic process. Some authors have used ethanol for mucilage precipitation, since this allows separation of specific components of mucilages, such as pectins (Cárdenas et al., 2008), and some low molecular weight components (Cárdenas et al., 2008). However, ethanol may be inconvenient for some components of the freeze‐drying device such as seals and O‐rings. The lyophilization process in those cases requires prior washing of the samples to remove ethanol, which also removes water soluble material and leads to lower yields. Thus, the average yield for the three ages in this work was ~20 g/kg of fresh nopal, which is significantly higher than the 6.2 g/kg of fresh cactus reported by Cárdenas et al. (2008).

### 2.2 Quantitation of sugar contents in nopal mucilages

Samples for the determination of sugar contents in the mucilages consisted of solutions of standard arabinose, fructose, galactose, glucose, rhamnose, and xylose at concentrations of 50 mg/ml in ultrapure Milli Q water (Millipore), which were diluted in the range from 0.19 to 50 mg/ml. With these solutions, standard or reference curves were obtained to determine the concentration of each sugar. Separately, 0.2 g of mucilage of each age was dispersed in 10 ml of Milli Q water at 40°C for 1 hr. Afterward, mucilage solutions were filtered using a Millipore Millex Syringe Filter unit of 0.45 µm (Merck) and stored at 4°C prior to analysis. Sugar contents in the mucilages were determined at 30°C by using an HPLC (LC‐MS 2020, Shimadzu) system consisting of a controller system CBM‐20, two binary pumps LC‐20AD, a degasifier DGU‐20A3R, an auto sampler SIL‐20AC, a column furnace CTO20A, a photodiode array detector UV‐Vis‐SPD‐M20A, a refractive index detector RID‐10A, a Prevail Carbohydrates ES 5u (250 × 4.6 mm) column operated at 30°C, and the software LabSolutions 5.0. The mobile phase was acetonitrile with ultrapure water (8:2 v/v) in isocratic elution at 1 ml/min and sample injection volume of 20 µl. Detection of components was performed at a wavelength (*λ*) of 195 nm. Sugars from the three mucilages were identified by comparison to standards of arabinose, fructose, galactose, glucose, rhamnose, and xylose and then quantitated by using their respective standard curves (Li et al., 2007).

### 2.3 Quantitation of uronic acids (pectic compounds) in nopal mucilages

Quantitation of uronic acids from the different mucilages in water solutions (2 mg/ml) was performed by the colorimetric method using carbazole (Dische, 1947). First, 20 mg of mucilage was dispersed in 10 ml of distilled water at 40°C for 1 hr. Then, the samples were stored for 24 hr at 4°C prior to hydrolysis. A mixture of 100 µl of the mucilage solutions with 6 ml of concentrated H_2_SO_4_ was put in a stainless steel reservoir with 3 L of cold water and then boiled in a water bath at 90 ± 1°C for 10 min. The mixture was then cooled at room temperature and added to 0.4 ml of an alcoholic solution of carbazole (0.1% v/v). The absorbance was measured after 30 min in an UV‐vis spectrophotometer (160A, Shimadzu) at *λ* = 530 nm, meanwhile the concentration was determined via a calibration curve of a standard galacturonic acid (solutions of 0.25–1 mg/ml in distilled water were used).

### 2.4 Determination of the molecular weight of nopal mucilages

The *M*_w_ of the mucilages and the second virial coefficient (*A*_2_) were determined at a temperature of 25°C by SLS (Litesizer™ 500, Anton Paar GmBH). The SLS technique was chosen because it allows the measurement of absolute *M*_w_ (Sperling, 2001) by:

(1) $$\frac{\mathrm{Kc}}{R_{\theta }}=\frac{1}{M_{w}}+2A_{2}c$$

where *c* is the concentration of mucilage in the solution, *K* is the optical constant, and *R_θ_* is the Rayleigh ratio. The *K* and *R_θ_* were calculated as follows (Sperling, 2001):

(2) $$K=\frac{2\pi^{2}}{\lambda^{4}N_{A}}{\left(n_{0}\frac{dn}{dc}\right)}^{2}$$

(3) $$R_{\theta }=\frac{\left(I_{s}‐I_{0}\right)n_{0}^{2}}{I_{T}n_{T}^{2}}R_{T}$$

In Equation 2, *λ* = 658 nm is the wavelength of the incident light, *N*_A_ is the Avogadro's number, *n_0_* = 1.33027 is the refractive index of distilled water, and d*n/*d*c* is the change of the refractive index as a function of concentration. In Equation 3, *I*_S_ and *I*_0_ are the scattered light intensities of solutions and solvent, respectively, *I*_T_ is the dispersed light intensity of a standard (toluene in this case), *n_0_* and *n*_T_ are the refractive indexes of water and toluene, respectively, and *R*_T_ = 1.14574 × 10^−5^ cm^−1^ is the Rayleigh ratio of toluene.

Four aqueous solutions of *T*, *Y*, and *M* nopal mucilages were prepared by mixing the appropriate amount of mucilage in distilled water. Then, each solution was filtered through #4 and #40 filter papers (Whatman) to retain coarse and gelatinous precipitates (20–25 µm) and fine particles (5–10 µm), respectively. The particle sizes of the mucilages in solution were investigated before index refraction and *M*_w_ determinations by dynamic light scattering measurements (Litesizer™ 500, Anton Paar). The obtained particle sizes varied between 0.33 and 7.22 µm. Filter papers with the retained mucilage components were dried and weighed to recalculate the final concentrations of mucilage solutions used for *M*_w_ determinations (Table 1). The refractive index as a function of mucilage concentration was determined by using a refractometer (Abbemat 550, Anton Paar) calibrated with toluene at 25 ± 0.05°C and *λ* = 589 nm. The time‐averaged intensity of scattered light of toluene, water, and mucilage solutions were measured at a scattering angle *θ* = 90°, and the *Kc/R_θ_* values of the solutions were calculated for each concentration of *T*, *Y*, and *M* mucilage by using Equations 2 and 3.

**TABLE 1:** Concentrations of diluted mucilage solutions for *M*_w_ determination using SLS and rheological measurements with different ages

Concentration of the mucilage solutions for *M*_w_ determinations		
*T* (g/cm^3^)	*Y* (g/cm^3^)	*M* (g/cm^3^)
0.00316	0.00309	0.00114
0.00474	0.00464	0.00273
0.00632	0.00619	0.0041
0.00948	0.00928	0.00546

Density of freeze‐dried mucilages (g/cm^3^)		Concentration of solutions for rheological measurements			
		wt.%	*T* (g/cm^3^)	*Y* (g/cm^3^)	*M* (g/cm^3^)
*T*	0.949	2	0.0201	0.0202	0.0202
*Y*	0.953	4	0.0406	0.0407	0.0407
*M*	0.963	6	0.0612	0.0616	0.0618

### 2.5 Preparation of mucilage solutions for rheological measurements

Mucilage aqueous solutions for rheological measurements were prepared at three different concentrations, 2, 4, and 6 wt.%, for each age (densities of freeze‐dried mucilages and concentrations of their solutions in g/cm^3^ are also included in Table 1). For this, each mucilage was dispersed in water by stirring at 300 rpm and heating at 60°C until a homogeneous solution was apparent (~40 min). Afterward, the solutions were filtered using a *#20* sieve (841 µm) and allowed to rest for 18 hr prior to rheological measurements. Finally, the pH of the solutions was measured using a potentiometer (pH 510 series, Oakton Instruments), which was calibrated with different buffers (J. T. Baker company); the pH of the solutions ranged between 6.0 and 6.3.

### 2.6 Rheological measurements in dynamic oscillatory shear flow

Rheological measurements for the solutions were performed under dynamic oscillatory shear flow by using a controlled stress rheometer (AR‐G2, TA Instruments) provided with a Peltier system for temperature control (±0.1°C) and a DIN concentric cylinders system (C–C) with *R_i_* = 14 mm, *R*_o_ = 15 mm, and *L* = 42 mm, where *R_i_* and *R*_o_ are the radius of the internal and external cylinders, respectively, and *L* the length of the internal cylinder. The measurements were performed in triplicate to assure reproducibility, at a temperature of 25°C, by using fresh samples in each case.

### 2.7 Statistical analyses

Statistical analyses were performed using SigmaPlot version 14.0 (Systat Software Inc.). The mean values for the variables studied in the different groups were compared by one‐way ANOVA followed by a Tukey multiple range test assuming that it has a significant difference with *p* ˂ .05.

## 3 RESULTS AND DISCUSSION

### 3.1 Quantitation of sugar contents in nopal mucilages

The contents of galactose, rhamnose, xylose, uronic acids, and other sugars in nopal mucilages are presented in Table 2. From this, it can be seen that *M* mucilage has the highest content of uronic acids or pectic compounds with 30.31 g for each 100 g of mucilage, which is significantly different from the content of the same compound in the other two mucilages (*p* ˂ .05). The content of uronic acids in the *T* and *Y* mucilages was similar to that found for nopal mucilage purified with isopropanol and smaller than the fraction of total pectic mucilage (60.66%) reported by Bayar et al. (2016). Also, *M* mucilage in this work exhibited a higher content of uronic acids than a different sample of purified *O. ficus‐indica* with 360 days of maturation (19.5%) reported by Trachtenberg and Mayer (1981), and also higher than mucilages from other *Opuntia* species (20%–25%), but lower than other subfamilies of *Cactaceae* (44%–51%), both reported by Saag et al. (1975). Table 2 also shows that the concentration of uronic acids increases with the age of the cladodes, which is consistent with reports for purified mucilage from young and mature cladodes of different varieties of *O*. *ficus‐indica* (Ribeiro et al., 2010). The increase in concentration of uronic acids and the decrease in galactose, rhamnose, and xylose contents with the age (see Table 2) may be related to increased biosynthesis of sugar precursors of homogalacturonans in mucilaginous cells, which are used to regulate the water content in the plant (Baba, 2006; Nobel et al., 1992).

**TABLE 2:** Concentration of sugars in *Opuntia ficus‐indica* with different ages

Sugar	*T*	*Y*	*M*
Arabinose	–	0.054 ± 0.001^b^	0.043 ± 0.001^a^
Fructose	0.13 ± 0.02^a^	0.38 ± 0.04^b^	0.30 ± 0.03^b^
Galactose	8.52 ± 0.12^c^	5.88 ± 1^b^	1.48 ± 0.13^a^
Glucose	3.26 ± 0.1^a^	16.54 ± 0.29^c^	9.77 ± 0.50^b^
Rhamnose	8.05 ± 0.44^c^	1.57 ± 0.17^b^	0.38 ± 0.09^a^
Xylose	19.22 ± 0.16^c^	13.37 ± 0.65^b^	1.24 ± 0.17^a^
Uronic acid	9.59 ± 0.48^a^	10.68 ± 0.61^a^	30.31 ± 4.27^b^

Note

Values represent average ± standard deviation in g/100 g of mucilage (*n* = 3). Results in rows labeled with different letters indicate significant differences for Tukey (*p* ˂ .05).

On the other hand, Table 2 shows a decrease in the total amount of neutral sugars (namely, arabinose, fructose, galactose, glucose, rhamnose, and xylose) with the age of the cladodes, being 39.18 g in *T*, 37.79 g in *Y*, and 13.17 g in *M* (the sum of all the above mentioned sugars) for each 100 g of mucilage. This result is consistent with a report by Ribeiro et al. (2010) regarding a lower concentration of neutral sugars and higher concentration of uronic acids in mucilages extracted from mature cladodes as compared to those found in young ones. In addition, Table 2 shows that there is a decrease in the concentration of galactose, rhamnose, and xylose along with increased age. A decrease or even the absence of neutral sugars, such as rhamnose, in mucilages from *Opuntia ficus indica* has been related to the presence of pectic compounds in the polymeric chain (Habibi et al., 2004). Other researchers have suggested that a low concentration of rhamnose, xylose, and galactose indicates the presence of long chains of molecules of homogalacturonans (uronic acids) that contribute to the formation of pectic compounds, which are essential for gelation of these biopolymers (Buchanan et al., 2015; Di Lorenzo et al., 2017; Goycolea & Cárdenas, 2003; Habibi et al., 2004).

The results in this section indicate that changes in sugar concentration in the mucilages along with age affect the physicochemical properties of nopal mucilages, which may be relevant, in particular, for their rheological behavior in solution and the critical concentration to form gels.

### 3.2 *M*_w_ of nopal mucilages and concentration regimes

**FIGURE 1:**
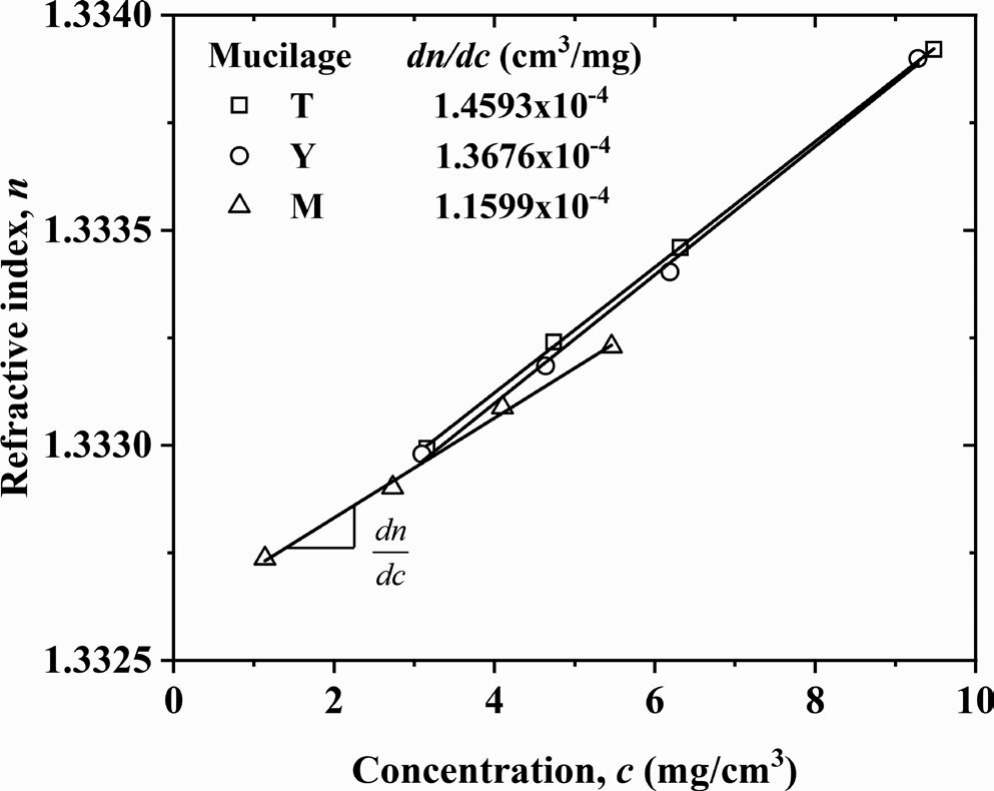
Refractive indexes of *T*, *Y* and *M* nopal mucilage solutions as functions of concentration. Continuous lines indicate the linear fittings

**FIGURE 2:**
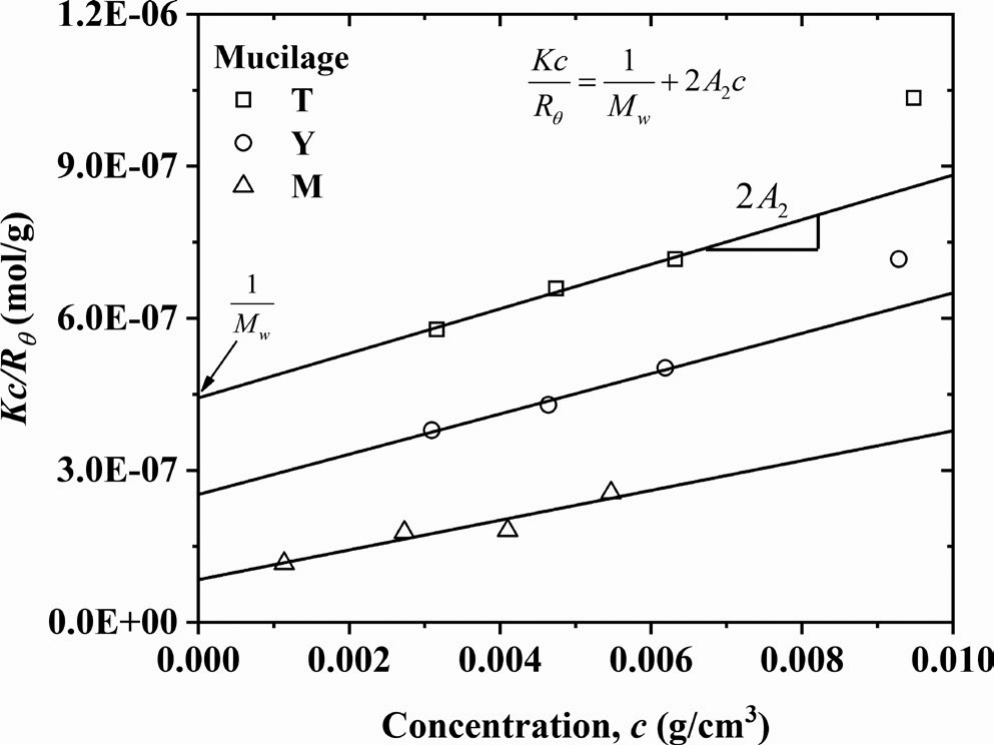
Kc/Rθ values as a function of concentration for *T*, *Y* and mature *M* nopal mucilage solutions. Continuous lines indicate the fitting to Equation 1

Measurement results of the refractive index as a function of mucilage concentration are presented in Table 3 and Figure 1, respectively. The slopes, d*n*/d*c*, for the different mucilages were obtained by linear fitting of the *n* versus *c* plots and the resulting values are inserted in Figure 1. Also, the *Kc/R_θ_* values are presented in Table 3 and are plotted versus the concentration of *T*, *Y*, and *M* mucilages in solutions in Figure 2. The continuous lines indicate the fittings to Equation 1 for the different ages. It is important to note here that the highest concentrations for *T* and *Y* mucilage solutions were not fitted by Equation 1, which indicates that these concentrations do not fall in the dilute regime. Finally, from the ordinate to the origin and the slope of each linear relationship, *M*_w_ and *A*_2_ were calculated for each nopal mucilage sample and the resulting values are reported in Table 4. It can be seen from this table that the lowest *M*_w_ corresponds to the mucilage from the *T* cladodes and that older ones result in increasing *M*_w_. On the other hand, for all the mucilages *A*_2_ was higher than 0, which indicates that water is a good solvent for this kind of mucilages. Positive values of *A*_2_ are mostly ascribed to repulsive interactions between biopolymer chains (Teraoka, 2002).

**TABLE 3:** Refraction indexes, *n*, and *Kc/R_θ_* values as a function of concentration, *c*, for nopal mucilage solutions with different ages

*c* (g/cm^3^)	*n*	*Kc/R_θ_* (mol/g)
*T*		
0.00316	1.33299	5.781 × 10^–7^
0.00474	1.33324	6.59 × 10^–7^
0.00632	1.33346	7.171 × 10^–7^
0.00948	1.33392	10.35 × 10^–7^
*Y*		
0.00309	1.33298	3.79 × 10^–7^
0.00464	1.33318	4.29 × 10^–7^
0.00619	1.3334	5.02 × 10^–7^
0.00928	1.3339	7.17 × 10^–7^
*M*		
0.00114	1.33274	1.164 × 10^–7^
0.00273	1.3329	1.781 × 10^–7^
0.0041	1.33309	1.813 × 10^–7^
0.00546	1.33323	2.558 × 10^–7^

**TABLE 4:** Molecular weight, *M*_w_, of nopal mucilages with different ages, second virial coefficient, *A*_2_, and concentration regimes, c*, of their solutions

Mucilage	*M*_w_ (× 10^6^ g/mol)	*A*_2_ (× 10^–4^ cm^3^‐mol/g^2^)	c*~(A_2_ *M*_w_)^−1^ (× 10^–3^ g/cm^3^)
*T*	2.258	0.229	20.13
*Y*	3.963	0.199	12.68
*M*	11.869	0.147	5.74

On the other hand, the rheological behavior of polymeric solutions is in general dependent on the concentration of macromolecules. Therefore, it is important to determine the different concentration regimes that may occur for the nopal mucilage solutions. The different concentration regimes may be determined in terms of the overlap concentration, c*, which was calculated by c*~(*A*_2_ *M*_w_)^−1^ (Teraoka, 2002). The overlap concentration, c*, is defined as the critical concentration at which the polymeric chains, assumed as random coils, overlap. For polymer concentration c < c* the solution regime is diluted. If c > c*, then the polymeric chains overlap and the solution is in the semidiluted regime. The values of the overlap concentrations calculated for the different mucilages are also reported in Table 4. From these data it is clear that increasing the age decreases the overlap concentration, as expected by increasing *M*_w_. Considering the c* values for the nopal mucilage samples with different ages, it is clear that all the solutions analyzed in this work lie in the semidiluted (c > c*) and concentrated (*c »* c*) regimes.

Finally, it is noteworthy from data in Table 4 that *M*_w_ values for the three mucilages are consistent with those reported by other authors for *O*. *ficus*‐*indica* mucilages obtained by freeze‐drying (Cárdenas et al., 1997; Majdoud et al., 2001; Trachtenberg & Mayer, 1981). In addition, these *M*_w_ values are of the same order of magnitude as those reported for other polysaccharides hydrocolloids as Xanthan and Guar gums (Lazaridou & Biliaderis, 2007; Petri, 2015; Quinzio et al., 2018) and suggest the use of nopal mucilages in similar rheological applications.

### 3.3 Linear viscoelastic behavior of nopal mucilage solutions

**FIGURE 3:**
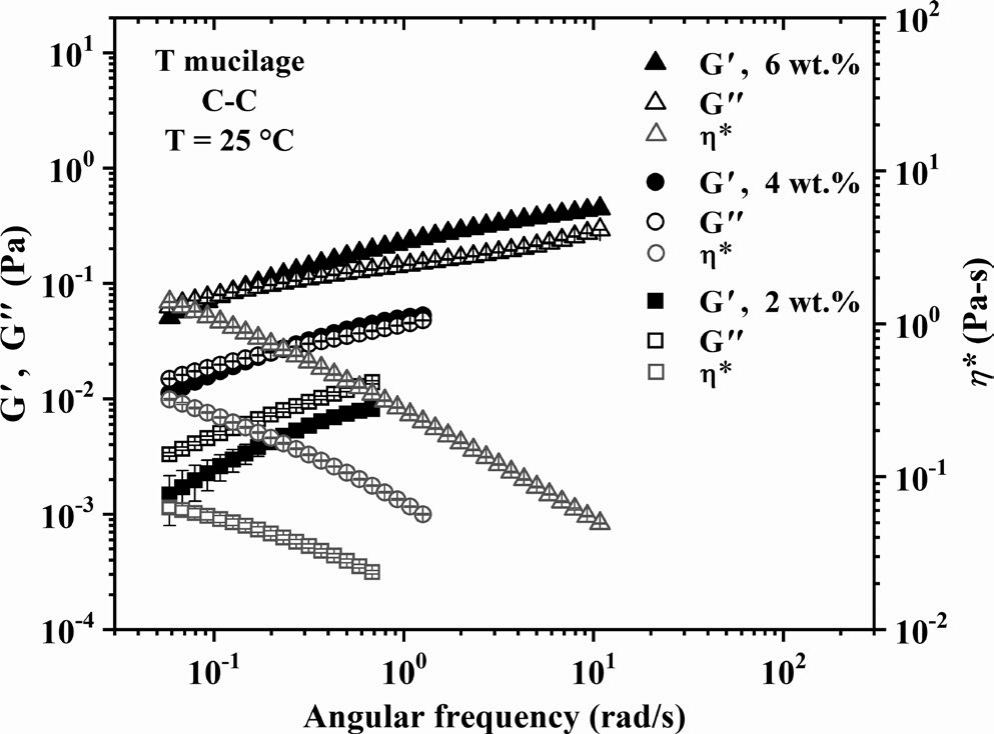
Linear viscoelastic behavior of *T* nopal mucilage solutions. Error bars represent the standard deviation of three different measurements for each sample

**FIGURE 4:**
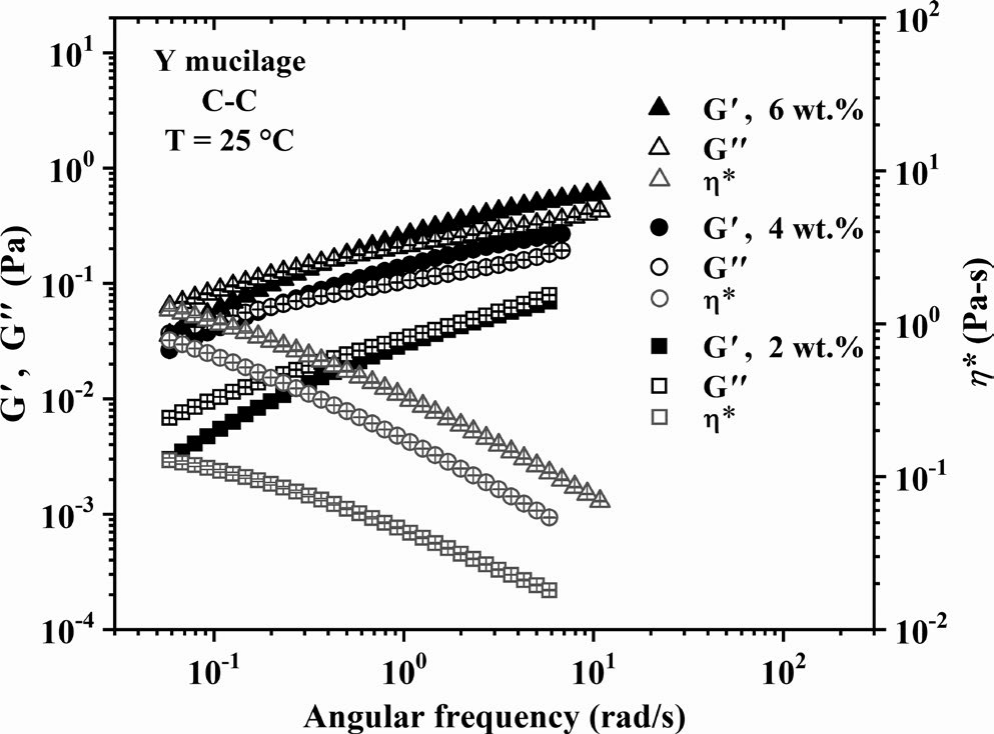
Linear viscoelastic behavior of *Y* nopal mucilage solutions. Error bars represent the standard deviation of three different measurements for each sample

**FIGURE 5:**
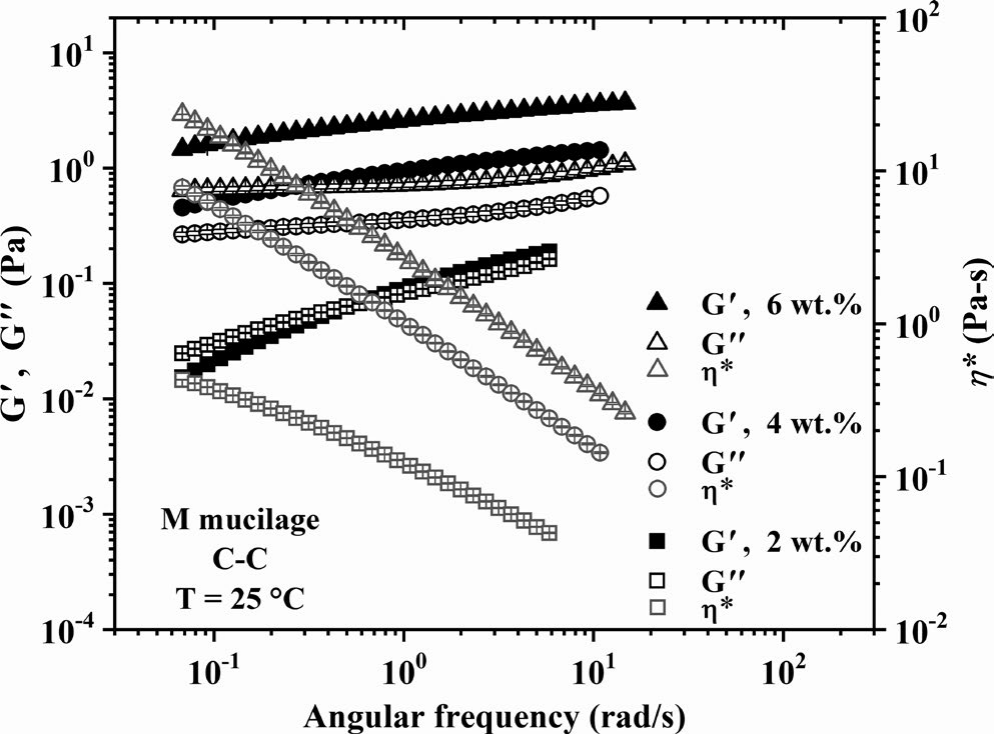
Linear viscoelastic behavior of *M* nopal mucilage solutions. Error bars represent the standard deviation of three different measurements for each sample

The linear viscoelastic behavior of the different mucilage solutions is presented in Figures 3, 4, 5 in terms of the loss (*G*″) and storage moduli (*G*′) and complex viscosity (η*) as functions of the angular frequency (*ω*). Error bars represent the standard deviation of three different measurements for each sample and vertical lines indicate the angular frequency at the crossover point, that is, *G*′ = *G*″, whose inverse represents the longest relaxation time (*λ*_R_) of the macromolecules. Figures 3, 4, 5 show that *G*′ and *G*″ values increase along with the mucilage concentration as well as with the age (that is, *M*_w_). Also, it is clear that the behavior of solutions changes from purely viscous (2 wt.% *T* in Figure 3), as evidenced by the predominance of the loss modulus, to viscoelastic (4, 6 wt.% *T* in Figure 3, 2–6 wt.% *Y* in Figures 4, and 2 wt.% *M* in Figure 5), as shown by the crossover point and increase in *λ_R_* with concentration and molecular weight. In particular, it is interesting to focus on data for 4 and 6 wt.% of *M* mucilage in Figure 5; note how *G*′ and *G*″ are almost parallel, with *G*′ > *G*″, and independent of the angular frequency, which is indicative of a gel‐like behavior (Aktas et al., 2014). The ratio *G*′/*G*″ for the 4 and 6 wt.% of *M* mucilage solutions (Figure 5) is of the order of 2.09 and 2.95, respectively, which indicates that *M* mucilages form weak gels (Khandal et al., 2019) at these concentrations. This gel‐like behavior is particularly interesting and will be further analyzed in the next section.

The results in the previous paragraph are in agreement with existing reports on the flow behavior of nopal mucilage solutions. Newtonian and non‐Newtonian shear‐thinning behavior has been reported for solutions of mucilages from cactus and other biopolymers, such as pectins, gums, and cellulose derivatives, depending on their concentration (Cárdenas et al., 1997; Contreras‐Padilla et al., 2016; de Vargas et al., 1993; León‐Martínez et al., 2011; Majdoud et al., 2001; Médina‐Torres et al., 2000; Morris et al., 1981; Pérez‐González et al., 1992; Trachtenberg & Mayer, 1982). According to Majdoud et al. (2001), mucilage macromolecules extracted from cladodes are flexible with some branches due to the presence of rhamnose in their structure. Thus, above c* mucilage solutions may exhibit a low‐shear Newtonian region characterized by a zero‐shear viscosity which appears enhanced with increasing the polymer concentration. Then, shear‐thinning occurs with increasing shear rate and concentration (see the η* values in Figures 3, 4, 5) due to disentanglement and orientation of the polymeric chains under shear. Therefore, the rheological behavior exhibited by the solutions with *T* (*M*_w_ = 2.258 × 10^6^ g/mol) and *Y* (*M*_w_ = 3.963 × 10^6^ g/mol) mucilages is fully consistent with that of semidiluted solutions (see the concentration regimes in Table 3). As for the *M* solutions (*M*_w_ = 11.869 × 10^6^ g/mol), higher concentration regimes are expected at low concentrations of mucilage due to the low c* value (see Table 3). Some authors have reported rheological gel‐like behavior for nopal mucilage solutions at concentrations higher than 3 wt.% (León‐Martínez et al., 2011; Médina‐Torres et al., 2000), in agreement with our observations for 4 and 6 wt.% of *M* mucilage solutions (Figure 5). This flow behavior is further explored below.

Gel‐like structures in this sort of mucilages result from different factors as intra‐ and intermolecular interactions, that is, entanglements and hydrogen bondings, respectively, sugar and pectic compound contents, and by the presence of Ca^2+^, Na^+^, and K^+^ ions and other minerals in nonpurified mucilages (Cárdenas et al., 2008; Majdoud et al., 2001), which control the degree of crosslinking. In this work, in which nonpurified mucilage has been utilized, Ca^2+^‐, Na^+^‐, and K^+^‐mediated crosslinks and physical intermolecular interactions play a key role in the observed gel‐like behavior. A proximate chemical analysis of these nopal mucilages samples (Muñoz‐López, 2016) shows that the amount of ashes was 20.81 ± 0.97 g/100 g, 28.19 ± 0.15 g/100 g, and 28.56 ± 0.29 g/100 g in the *T*, *Y*, and *M* mucilages, respectively, whose major part corresponds to ions. This increase in ion concentration as well as the enhancement of physical interactions, that is, entanglements, with increasing molecular weight and age, give rise to the gel‐like behavior with increasing mucilage concentration. In addition, gelation of mature mucilage systems (4 and 6 wt.%) is related to its high concentration of uronic acids or pectic compounds (see Table 2), which are known to be precursors of gel‐like structures in this sort of mucilages (Cárdenas et al., 2008; Goycolea & Cárdenas, 2003; Habibi et al., 2004).

**FIGURE 6:**
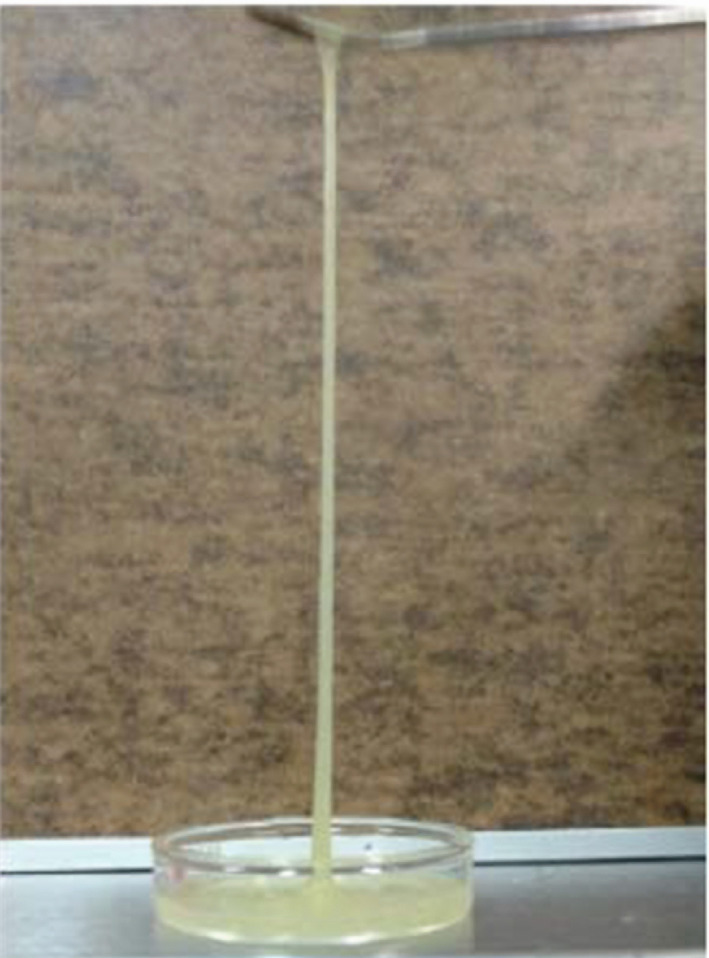
Picture of the solution with 6 wt.% of *M* mucilage subjected to elongation and showing its state of high elasticity

To conclude this section, it is worth emphasizing the increase in shear viscosity and elasticity of the solutions by increasing the age of the mucilage for a given concentration (see the moduli and η* values in Figures 3, 4, 5), which is fully in agreement with the increase in *M*_w_ along with the age. Considering the large size of nopal mucilage macromolecules, as expected by their high *M*_w_, their flexible conformation and the concentration regimes lying above c*, highly elastic solutions are expected to be formed by this kind of mucilages, in particular by *M* mucilage. Elasticity of a fluid may be informally understood as its ability to form threads. Figure 6 displays a picture of the solution with 6 wt.% of *M* mucilage subjected to elongation and showing its state of high elasticity. This characteristic of nopal mucilage solutions enables their use as binders in films and coatings.

Finally, it may be concluded that linear viscoelastic properties of nopal mucilages are akin to those reported for other hydrocolloids. Overall, the molecular weights and rheological behaviors reported in this work for nopal mucilages are comparable to those of widely used thickeners such as xanthan and guar gums. Subject to performing their analysis in steady shear and elongation flows, at this point we suggest that nopal mucilages may be safely used as thickening agents or rheological modifiers to improve textural or sensory characteristics in a variety of applications. The results in this work may be used as a guide to select its possible application regarding their age.

## 4 CONCLUSIONS

Changes in molecular characteristics of nopal mucilages with their age and its influence on the rheological behavior of their solutions were analyzed in this work by light scattering, HPLC and linear viscoelastic measurements. From the results in this work, it can be concluded that:

- The molecular weight (*M*_w_) of the mucilages increases along with the age.
- Concentration of uronic acids increases with age, meanwhile galactose, rhamnose, and xylose contents decrease.
- The rheological behavior of nopal mucilage solutions depends on the age of the cladodes. Increasing *M*_w_ with age results in enhanced viscoelastic behavior of solutions, namely, higher viscosity, higher elasticity, and gel‐like behavior at lower mucilage concentrations. Besides, decrease in neutral sugar contents and increase of pectic compounds (uronic acids) in mucilages with age promotes formation of gel‐like structures.
- Solutions of tender, young, and 2 wt.% of *M* mucilages exhibited a shear‐thinning behavior. However, solutions with 4 and 6 wt.% of *M* mucilages showed weak gel‐like behavior.
- Linear viscoelastic properties of nopal mucilages are akin to those reported for other hydrocolloids. Therefore, nopal mucilages may be safely used as thickening agents or rheological modifiers to improve textural or sensory characteristics in a variety of applications. The results in this work may be used as a guide to select its possible application regarding their age.

## CONFLICT OF INTEREST

The authors declare that they have no known competing financial interests or personal relationships that could have influenced the work reported in this paper.

## AUTHOR CONTRIBUTIONS

**Francisco Rodriguez‐Gonzalez:** Conceptualization (lead); Data curation (equal); Formal analysis (equal); Investigation (equal); Methodology (equal); Validation (equal); Writing‐original draft (lead); Writing‐review & editing (lead). **Jose Perez‐Gonzalez:** Conceptualization (lead); Data curation (lead); Formal analysis (lead); Investigation (equal); Methodology (equal); Supervision (lead); Validation (equal); Writing‐original draft (lead); Writing‐review & editing (lead). **Cesar Nadem Muñoz‐Lopez:** Data curation (equal); Formal analysis (equal); Investigation (equal); Methodology (equal); Validation (equal). **Silvia Viridiana Vargas‐Solano:** Data curation (equal); Formal analysis (equal); Investigation (equal); Methodology (equal); Validation (equal). **Benjamin Marcos Marin‐Santibañez:** Data curation (equal); Formal analysis (equal); Investigation (equal); Methodology (equal); Validation (equal).

## Data Availability

The raw/processed data required to reproduce these findings are available upon request.
